# Commentary on the surgical outcomes of glioblastoma multiforme in low and middle-income countries: current state and future directions

**DOI:** 10.1097/MS9.0000000000002804

**Published:** 2024-02-12

**Authors:** Emmanuel Gudu, Noor Salyani, Peter Nganga Wambu, Bartay Matui

**Affiliations:** a Moi University, School of Medicine, Kenya

In LMICs, the diagnosis of glioblastoma multiforme (GBM) is viewed as a death sentence. Yet, despite the immense challenges, the desire for better outcomes prevails. The article delves into the realities of the challenges faced in managing this aggressive brain tumor. One of the strengths of the paper is its extensive review of the existing literature, highlighting the significant gaps in research and the disparities in healthcare infrastructure between LMICs and high-income countries (HICs)^[1]^. The authors underscore the multifaceted challenges exploring the lack of an efficient healthcare framework^[2]^. However, the paper also has limitations such as language bias and heterogeneity exhibited by the studies referenced^[1,2]^.

To address the challenges faced by LMICs, the article suggests solutions such as improving healthcare infrastructure, increasing access to advanced medical facilities, and enhancing training for healthcare professionals. Furthermore, advocating for policy changes to secure funding for research and treatment can help bridge the gap in GBM management.

The article also notes that patients in HICs often benefit from advanced surgical techniques and technologies, leading to better survival rates while LMICs struggle with limited resources, resulting in varied surgical outcomes and lower overall survival rates. Delayed diagnosis plays a major role in worsening surgical outcomes leading to poorer prognosis and quality of life^[2]^. The introduction of affordable alternatives, such as intraoperative neurosurgery ultrasound, is a promising step toward improving surgical precision and patient outcomes in LMICs. While other forms of therapies arise such as anticancer stem cell therapy and immunotherapeutic Strategies^[3,4]^, they remain out of reach for the majority of the population in LMICs.

The article also calls for collaborative efforts to enhance surgical management and improve patient care in these resource-limited settings while factoring key concerns such as cost of treatment to improve outreach.

## Data Availability

Not applicable. Data availability statement was not required for this correspondence.

## References

[1] Muili AO Aderinto N Ayodeji A. Surgical outcomes of glioblastoma multiforme in low and middle-income countries: current state and future directions. Ann Med Surg. 2024;86:5326–5333. 10.1097/MS9.0000000000002362 PMC11374186 39239018

[2] Tini P Rubino G Pierpaolo P. Challenges and opportunities in accessing surgery for glioblastoma in low-middle income countries: a narrative review. Cancers (Basel). 2024;16:2870–0. 39199641 10.3390/cancers16162870 PMC11352297

[3] King JL Benhabbour SR. Glioblastoma multiforme – a look at the past and a glance at the future. Pharmaceutics. 2021;13:1053. 34371744 10.3390/pharmaceutics13071053 PMC8309001

[4] Rong L Li N Zhang Z. Emerging therapies for glioblastoma: current state and future directions. J Exp Clin Cancer Res. 2022;41:142. 35428347 10.1186/s13046-022-02349-7 PMC9013078

